# Cognitive processes in autism: Repetitive thinking in autistic versus non-autistic adults

**DOI:** 10.1177/13623613211034380

**Published:** 2021-07-22

**Authors:** Kate Cooper, Ailsa Russell, Steph Calley, Huilin Chen, Jaxon Kramer, Bas Verplanken

**Affiliations:** University of Bath, UK

**Keywords:** autism, cognition, repetitive behaviours and interests

## Abstract

Repetitive and restricted behaviours are a core feature of autism, and cognition in autistic individuals may also be repetitive and restricted. We aimed to investigate the relationship between repetitive behaviours and repetitive thinking. We predicted that autistic people would experience more repetitive, perseverative, visual and negative cognition than controls. We predicted that repetitive thinking would be associated with repetitive behaviours in the autistic participants. We recruited autistic (*n* = 54) and control (*n* = 66) participants who completed measures of insistence on sameness and obsessive-compulsive behaviours. Participants then took part in 5 days of descriptive experiencing sampling, recording their thoughts when a random alarm sounded. Consistent with our hypothesis, autistic participants reported more repetitive thinking. Contrary with our other hypotheses, autistic participants reported equivalent frequency of perseveration, visual thoughts and negative thoughts to non-autistic participants. Moreover, participants who reported more obsessive thinking reported more repetitive behaviour (insistence on sameness), but there was no such relationship between repetitive thinking and behaviour. Autistic participants who reported more repeated thoughts in the descriptive experience sampling had significantly lower obsessive thinking scores. We conclude that anxiety focused cognitions may drive insistence on sameness behaviours, and that the relationship between repetitive cognition and behaviour is complex and warrants further investigation.

**Lay abstract:**

A core feature of autism is the tendency to do the same activity or behaviour repetitively. We wanted to find out if autistic people also experience repetitive thinking, for example, having the same thoughts repeatedly. We thought that there would be a link between repetitive behaviour and repetitive thinking. We asked 54 autistic people and 66 non-autistic people to complete questionnaires measuring repetitive behaviours and obsessive thinking. Next, participants were trained by a researcher to record their thoughts using a structured paper form. They then completed 5 days of thought recording, which they did each time a random alarm sounded on their mobile phone. We found that autistic people had more repetitive thoughts than non-autistic people, but they did not report having more negative or visual thoughts compared with non-autistic people. Autistic people who had more repetitive thoughts during the 5 days of thought recording did not report more repetitive behaviour. However, autistic people who reported more obsessive thinking, for example, more negative and unwanted thoughts, also reported higher levels of repetitive behaviour. We conclude that some repetitive behaviours may be linked to anxiety and that more research is needed to better understand repetitive behaviours in autism.

Repetitive, restricted behaviours, interests and activities are a core feature of Autism Spectrum Disorder (ASD), as outlined in the *Diagnostic and Statistical Manual of Mental Disorders* (5th ed.; *DSM*–5; American Psychiatric Association, 2013). This feature of autism encapsulates a wide range of phenomena, broadly conceptualised as ‘higher order’ and ‘lower order’ behaviours. Higher order behaviours are often referred to as insistence on sameness, that is, a preference for routine and ritualised behaviours, and lower order as repetitive sensory motor behaviours (Bishop et al., 2013). Studies have established an association between the latter and a general developmental delay, while insistence on sameness behaviours is specific to autism (Harrop et al., 2014).

Little is known about the internal drivers for behaviours which are restricted, that is, limited in range, and repetitive, that is, done multiple times. While they have been behaviourally defined, higher order restricted behaviours and interests reflect repetition and restriction at the conceptual level such as a preference for routine or a circumscribed interest. For example, an intense interest in a topic, pursued to the exclusion of other activities, is presumably evidence of significant, repetitive thinking on this interest. Another factor to consider is the evidence for high rates of co-occurring autism and Obsessive-Compulsive Disorder (OCD; Lai et al., 2019). Clinical and experimental research has sought to ensure a careful delineation of restricted and repetitive behaviour-related internal content and the intrusive, unwanted obsessional thoughts characteristic of OCD (e.g. Russell et al., 2013). While some studies find an overlap between repetitive behaviours and OCD compulsions at least in behavioural terms, OCD obsessional thoughts have been reported as distinct and separate from autism features in a network analysis (Ruzzano et al., 2015). Thus, the internal phenomenology of repetitive behaviours and restricted interests remains poorly understood in respect of content and process. Therefore, in this study we characterise repetitive and restricted thinking as part of the features of autism, and measure obsessional thinking under the assumption that it is separate from these autism features.

There is evidence that autistic people may experience more visual cognitive processing compared with the general population. Executive functioning refers to a higher order set of cognitive processes which are central to goal-directed behaviour, for example planning, attention and working memory. Previous research has found differences in executive functioning between autistic and non-autistic individuals (see Demetriou et al., 2018 for a review). In typical development, executive functioning tasks have been found to be verbally mediated from the age of 7 years (Hitch et al., 1989). In autistic people, there is evidence that many of these tasks are visually, rather than verbally mediated (for a review see Williams et al., 2016). Evidence from a preliminary study of introspection in autistic people suggested that this group are more likely to think in images than non-autistic people, who report predominantly verbal thoughts (Hurlburt et al., 1994). These studies point towards a different experience of cognition for autistic people, in terms of lowered use of verbal thoughts to mediate cognitive tasks, and a phenomenological experience which is more visual than verbal. One of the aims of this study was therefore to investigate the phenomenological experience in autistic people regarding the frequency of visual compared with verbal thoughts.

To measure the cognitive experience in autistic people, in this study we use a modified Descriptive Experience Sampling Method (DESM). This methodology has been used to capture in the moment experiences of typical and atypical or clinical phenomena across a wide range of populations (Lapping-Carr & Heavey, 2017). This method involves participants being randomly alerted to complete a structured record of their cognitive experiences at the time the alert happened. The DESM can be adapted to address the difficulties with measuring cognition in autistic people. This method has been used with autistic participants in the past; originally by Hurlburt et al. (1994), who used the method with three adults with Asperger’s syndrome and found that participants reported more visual thinking than non-autistic people. Hintzen et al. (2010) used descriptive experience sampling (DES) with 8 autistic participants and 14 controls, measuring their thoughts, mood, current social context and activities. This provided insights into the social preferences and motivation of autistic participants. Previous research has therefore found this method to be feasible with autistic participants (Chen et al., 2016; Hare et al., 2016; Hintzen et al., 2010; Kovac et al., 2016) and has provided an insight into the inner world of participants which could not have been acquired through a cross-sectional design.

In sum, autistic people engage in repetitive, restricted behaviours. It is possible that their thinking style is also repetitive and restricted; however, no research to date has directly investigated this.

## Aims

We aimed to further our understanding of repetitive and restricted patterns of activities, interests, and behaviours in autism by investigating related cognitive processes. We did this by adapting the method of DES to ensure an accessible recording of cognition in autistic adults.

We further aimed to compare thought content in autistic people versus non-autistic people to answer questions about restriction in thinking. We aimed to control for the potential confound of intrusive unwanted thoughts characteristic of OCD. Our main question was: Is the cognitive experience in autism repetitive, restricted and stereotyped, in line with behavioural features of autism?

## Hypotheses

1. Autistic participants will report having the same thoughts more frequently (repetitive thinking style) than non-autistic participants.
2. Autistic participants will report more restricted thought content, that is, a reduced number of thought categories (restricted thinking style) compared to non-autistic participants.
3. Autistic participants will report having thoughts rated as more negative in content than non-autistic participants.
4. Autistic participants will report having more visual thoughts than non-autistic participants.
5. Total number of repetitive thoughts will be positively associated with insistence on sameness behaviours in the autism group.

## Method

### Participants and design

Adults over the age of 18 years were invited to take part in the study. A total of 120 participants took part, 54 with a validated diagnosis of an Autism Spectrum Disorder from a professional and 66 without autism (controls). The obtained sample sizes allowed to reliably observe medium-size correlations within each group with alpha set at 0.05 and accepting a power of 0.80, and medium to large effect sizes with respect to comparisons between the groups. Participants were included in the autism group if they provided evidence of a clinical diagnosis of ASD, for example, a clinic letter confirming their autism diagnosis. All participants had to have access to a smartphone to take part. See Table 1 for a summary of demographic characteristics of participants. Participants needed to have the cognitive ability to read the information sheet and give informed consent, as well as to be able to read and respond to the questionnaire measures and complete the DES measures.

**Table 1.** Demographic variables.

Characteristic	Autism group		Control group		Full sample		Significant difference?
	*n*	%	*n*	%	***n***	**%**	
Gender							χ^2^(1,117) = 4.62 *p* = .032
Female	20	37	38	58	58	48	
Male	32	59	27	41	59	49	
Other	2	4	1	2	3	3	
Ethnicity							χ^2^(2,119) = 0.20, *p* = .904
White	50	94	62	94	112	94	
Asian	1	2	2	3	3	3	
Mixed race	2	4	2	3	4	3	
Relationship status							χ^2^(3, 120) = 7.75, *p* = .051
Single	30	56	26	39	56	47	
Cohabiting/married	14	26	19	29	33	28	
In a relationship	8	15	21	32	29	24	
Prefer not to say	2	4	0	0	2	2	
Highest educational level							χ^2^(4,119) = 11.56, *p* = .021
No qualifications	0	0	6	9	6	5	
GCSE/ A level	22	41	15	23	37	31	
Undergraduate degree	15	28	16	25	31	26	
Postgraduate degree	14	26	27	42	41	35	
Other	3	6	1	2	4	3	
Employment							χ^2^(2,119) = 1.79, *p* = .408
In education	18	34	24	36	42	35	
Employed	28	53	38	58	66	56	
Unemployed	7	13	4	6	11	9	
Age	*M*	*SD*	*M*	*SD*	*M*	***SD***	*t*(117) = 0.75, *p* = .456
	33.78	14.32	31.95	12.69	32.77	13.41	
	Min	Max	Min	Max			
	18	71	19	69			

*SD*: standard deviation; GCSE: General Certificate of Secondary Education.

Participants were recruited via online social media websites such as Twitter, Facebook and Reddit, and via posters and advertisements left in community centres and other public spaces.

**Figure 1.**
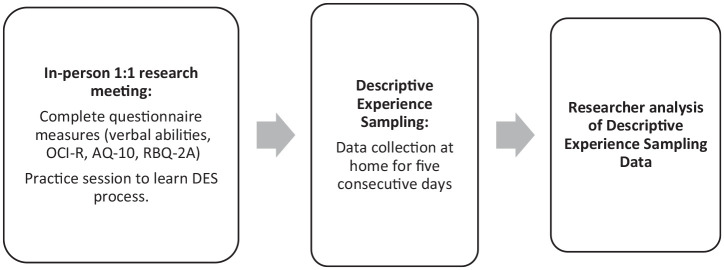
Procedure flowchart.

Participants completed consent and baseline questionnaires, followed by 5 days of DES (see Figure 1).

### Measures

#### Verbal abilities

Verbal abilities were assessed in all participants using the vocabulary sub-test of the Wechsler Abbreviated Scale of Intelligence (WAIS–IV). This assessment was used to match the two groups on verbal IQ. This ensured differences between groups in cognitions were not a result of verbal abilities.

#### Obsessive Compulsive Inventory–Revised (OCI-R)

The OCI-R is an 18-item self-report measure of the symptoms of obsessive-compulsive disorder, with items such as ‘I feel I have to repeat certain numbers’, ‘I find it difficult to control my own thoughts’ and ‘I collect things I don’t need’ (Foa et al., 2002). These items are scored on a 5-point Likert-type scale (0–4), indicating increasing frequency (α = 0.93). The scale has been found to be reliable and valid (Foa et al., 2002). The scale has been previously used with autistic populations (e.g. Russell et al., 2013), and the overall scale and subscales have good internal consistency and discriminant validity (Cadman et al., 2015). In this study, we report the total OCI-R score and obsessing subscale only, in line with previous research (e.g. Gay et al., 2011), as obsessing is the subscale most closely linked with repetitive thinking. The obsessing subscale is made up of three items, for example, ‘I frequently get nasty thoughts and have difficulty in getting rid of them’ with higher scores indicating greater frequency and intensity of obsessional thoughts.

#### Autism Quotient-10 (AQ-10)

This questionnaire is a 10-item screening measure for ASD (α = 0.85) (Baron-Cohen et al., 2001). Participants are asked to rate how strongly they agree or disagree with the statements using a 4-point Likert-type scale, for example, ‘I find it difficult to work out people’s intentions’, ‘I often notice small sounds when others do not’ and ‘I know how to tell if someone listening to me is getting bored’. A cut-off score of 6 is typically used to distinguish those who are likely to meet diagnostic criteria for ASD (Allison et al., 2012).

#### The Adult Repetitive Behaviours Questionnaire (RBQ-2A)

The RBQ-2A is a self-report measure of repetitive behaviours with 20 questions such as ‘Do you insist that aspects of daily routine must remain the same?’, ‘Do you insist on doing things in a certain way or re-doing things until they are “just right”?’, and ‘Do you insist on eating the same foods, or a very small range of foods, at every meal?’ with 3- or 4-point Likert-type scales indicating severity (Barrett et al., 2015). Autistic people score significantly higher on the measure than non-autistic individuals, suggesting that it is a valid measure of repetitive behaviours (Barrett et al., 2015). The 10-item insistence on sameness (IS) subscale was administered which measures more abstract repetitive behaviours such as routines or special interests (α = 0.90).

#### Descriptive Experience Sampling (DES)

DES techniques were adapted for this study to measure cognition. Participants downloaded an alarm app onto their smartphones. The app sent five alerts at random time points within an agreed 8-hr period each day for five consecutive days (*n* = 25 thought recording reminders). The alarms reminded participants to complete a written DES record designed for this study. The form prompted the participant to record the following information:

- What are you thinking? (Written response or drawing accepted)
- Is the thought visual, verbal or other? (Tick box response)
- How positive or negative is the thought? (−10 to +10 positivity rating on a visual analogue scale)

It was possible for participants to rate their thoughts in multiple categories, for instance, a single thought could be rated as ‘visual’, ‘verbal’, and ‘other’ if this is how it was experienced by the participant.

Thoughts recorded in the DES booklets were subject to content analysis. Coders followed a comprehensive codebook developed for this study to code each thought. A subset of coding was rated by a second researcher to ensure the process was reliable. The codebook was developed through a series of meetings with the research team. The adapted DESM method used in the present study did not include an expositional interview and relied on objective coding of recordings of thought made in the moment. However, we aimed to maintain an idiographic as well as nomothetic approach to the data. As the research question was primarily about repetition, the focus of coding was the content of the inner experience rather than form, although we included some aspects of previously reported typologies (Hurlburt & Heavey, 2006) in our coding system.

In terms of an idiographic approach to coding thought content, each record was allocated a keyword which summarised the main theme of the thought. This keyword was derived solely from the participant’s report and was selected as most representative of the thought content. This content analysis relied on the coder’s ability to interpret the content of a thought as a whole, and to do so objectively, without bias. The coder derived the core topic or focus found within the reported thought. The coder was directed to strike a balance between specificity and breadth, accurately capturing the focus of the thought, and encouraged to return to the keywords and amend them should a pattern emerge within each individual’s records. Thoughts were counted as repetitive when the same keyword was noted within the individual’s 25 DES records.

A top-down coding system drawing on previously reported categorisations of inner experience (i.e. emotion and multiple awareness) and categorisations potentially relevant to the study of thought content in autism was developed. These categories represented degree of introspection (metacognition), whether the thought was related to self/others/object(s) and whether a thought represented an action or ‘doing’. A single thought could score across multiple coding categories. In this way, each record was allocated a score relating to the following thought categories: meta-cognitive awareness; multiple awareness; tense (past, present, future); self-reference; other-directed; non-person oriented; action; emotion. Metacognitive awareness, defined as awareness of mental states, was coded for each entry as being either 1–3, with 1 indicating no metacognitive awareness and 3 indicating the highest level of metacognitive awareness. Multiple awareness, defined as the thought including two or more unrelated processes, was scored as being present or absent (0 or 1). Self-referent thoughts focused on the individual reporting the thought (0 or 1). Other-directed thoughts included another person (0 or 1). Non-person oriented thoughts included concrete objects or items (0 or 1). Action thoughts involved doing something (0 or 1). Emotion thoughts included a feeling or emotional state (0 or 1).

Restricted thinking was calculated by summing the total number of thought categories for each participant giving an index of restriction i.e. limited categories of thinking (see Table 2). We excluded the categories of metacognitive and multiple awareness from these summary scores as these codings were not binary, and represented thinking processes rather than content.

**Table 2.** Description of variables taken from the descriptive experience sampling data.

Number of repeated thoughts (repetition)	Number of thoughts repeated by each participant, that is, the number of times a keyword describing a particular thought was repeated.
Number of thought categories (restriction)	Number of thought categories applied to each participant’s data, that is, how many of the five categories (self-referent; other-directed; non-person oriented; action; emotion) were used to describe the 25 thoughts in each participant’s DESM data.
Positivity rating of thoughts	Mean of participant ratings from −10 to +10 of their perception of the negativity or positivity of each thought recorded in the DESM.
Percentage of visual thoughts	Percentage of thoughts reported in the DESM marked as ‘visual’ rather than ‘verbal’ or ‘other’. Note that thoughts could be marked as belonging to multiple categories.

DESM: Descriptive Experience Sampling Method.

Participants had rated their own thoughts within the thought recording process. We used the individual’s own description of their thoughts, calculating the dependent variables of percentage of reported visual thoughts, and mean positivity rating (−10 to 10).

The initial coding framework was subject to try-out and refinement. Two members of the research team independently coded the first five participants using the coding framework with inter-rater agreement of 74%. This allowed the framework to be refined to ensure greater reliability, primarily related to the definitions for ‘meta-cognitive awareness’. A random sample of 20% of the final thought booklets were coded by a second independent rater. The final coding framework demonstrated excellent inter-rater reliability with an intraclass correlation coefficient (ICC) = .957 (95% CI .950–.964).

### Procedure

Potential participants were provided with an information sheet about the study, which included information about what participation will entail, alongside an explanation of the overall study principles. The information sheet was adapted for the needs of autistic participants, with short, clear sentences and visual information where appropriate.

Autistic participants were asked to show a letter from a healthcare professional confirming their autism diagnosis. Participants gave informed consent and then completed the self-report, standardised measures and the brief measure of verbal abilities (see Figure 1). Next, participants were supported in setting up the reminder app on their smartphone and trained in the completion of the DES form. A ‘practice’ form was developed for this purpose and was used by the researcher to guide participants through the process of reporting their thoughts in the DES form. The practice form gave participants the opportunity to record their thoughts in response to the app alarm with the support of a researcher. Participants were also offered remote support by a researcher for the duration of the 5 days in which they were recording their thoughts. On completion of the experimental procedure (i.e. five days with five daily thought recordings) participants returned the DES booklets using prepaid envelopes.

All participants attended the training, and 80% of these returned the DES booklets (*n* = 96), comprising 44 of the autistic participants and 52 controls. Following participation, a debrief sheet was emailed to all participants which gave further information about the study. Participants were reimbursed for their time with a £10 shopping voucher.

### Ethical considerations

All participants were provided with an information sheet and if willing, completed a consent form. Participants were aware of their right to withdraw from the study. A distress protocol was established in case participants experienced any psychological distress arising from participation in the study.

### Community involvement

Five autistic adults were consulted about the study materials and procedure during a pilot study to ensure clarity and accessibility.

## Results

We conducted preliminary qualitative analysis to categorise each of the records from the DES booklets. See Table 3 for the overall findings from this analysis by group. The most frequently reported thought category for both groups was thoughts involving an action, with 65% of thoughts reported by autistic participants in this category and 68% of control participants. The next most common category was thoughts referencing the self, in 57% of the thoughts reported by autistic participants and 59% by control participants. The least common category was thoughts involving multiple awareness, reported in 9% of the samples from autistic participants and 3% from controls.

**Table 3.** Mean score and *SD*s, and percentages for categorisation of thoughts recorded through descriptive experience sampling.

Group	Metacognitive awareness	Multiple awareness	Self-reference	Other-directed	Non-person oriented	Action	Emotion
	*M* (*SD*)	%	%	%	%	%	%
Autism	1.62 (0.35)	9	57	24	31	65	22
Control	1.60 (0.32)	3	59	23	26	68	11

*SD*: standard deviation.

To test hypotheses 1–4, we conducted a one-way analysis of variance with the independent variable group ( autism versus control) and dependent variables: number of repeated thoughts (i.e. repetitive thinking), number of thought categories (i.e. restricted thinking), positivity rating of thoughts and percentage of thoughts reported as visual (see Table 4). In line with hypothesis one, autistic participants reported significantly more repeated thoughts than controls *F*(1,94) = 11.29, *p* = .001 (η^2^ = .11). We did not find support for hypothesis 2 as there were no significant differences between autistic participants and controls in restricted thinking style, that is, number of categories of thought did not differ between the groups, suggesting repetitive rather than restricted thinking. Hypothesis 3 was not supported since there was no difference between autistic participants and controls in positivity ratings of thoughts. There was no support for hypothesis 4 as there was no difference between groups between the number of visual thoughts reported.

**Table 4.** Means and standard deviations (*SD*) for Autism group (*n* = 54), Controls (*n* = 66) and results of one-way ANOVA.

Measure	Autism		Control		*F*	η^2^
	*M*	*SD*	*M*	*SD*		
DESM–Number of repeated thoughts^a^	10.07	3.34	7.87	3.08	11.29**	.11
DESM–Number of thought categories	4.81	0.39	4.65	0.56	2.71	.03
DESM–Positivity rating of thoughts	0.78	1.79	1.11	1.36	1.00	.01
DESM–Percentage of visual thoughts	0.32	0.28	0.34	0.26	0.11	.00
Insistence on sameness	2.28	0.45	1.42	0.34	142.64***	.55
Obsessive Compulsive Inventory	27.85	14.02	9.91	8.45	74.93***	.39
Verbal abilities	12.42	3.20	11.53	2.37	2.71	.03
AQ-10	7.31	1.99	2.38	2.18	164.47***	.58

ANOVA: analysis of variance; DESM: Descriptive Experience Sampling Method; AQ-10: Autism Quotient-10; OCI-R: Obsessive Compulsive Inventory–Revised.

* *p* < .05. **p *<* .01. ***p *<* .001.

a This analysis was also conducted controlling for OCI-R score, and remained significant *F*(1, 93) = 9.82, *p* = .002.

We conducted bivariate Pearson correlations between all study variables by group, to test hypothesis 5 (see Table 5). There was no significant association between insistence on sameness scores and number of repeated thoughts in either group.

**Table 5.** Correlations between variables for autistic and control participants.

		Autistic participants (below the diagonal) and Control participants (above the diagonal)								
		1.	2.	3.	4.	5.	6.	7.	8.	9.
1.	DESM–Number of repeated thoughts	–	0.13	0.08	0.11	0.11	−0.02	−0.15	−0.21	0.02
2.	DESM–Number of thought categories	0.14	–	0.02	−0.27	−0.11	−0.05	0.02	0.06	−0.16
3.	DESM–Positivity rating of thoughts	.14	0.06	–	.29*	−.15	−.17	−0.23	−.03	−.04
4.	DESM–Percentage of visual thoughts	.13	0.13	.21	–	.02	.08	0.02	.11	.02
5.	Insistence on Sameness	−.06	0.04	−.42**	.13	–	.67***	.32**	−.14	.34**
6.	Obsessive Compulsive Inventory	−.13	−0.15	−.36*	.04	.67***	-	.75***	−.20	.41**
7.	OCI-R Obsessing	−.31*	−0.07	−.32*	0.05	.46**	.65***	–	−.04	.30*
8.	Verbal abilities	.04	0.14	−.19	.26	.11	−.09	−.02	–	.05
9.	AQ-10	.04	−0.15	−.35*	−.07	.50***	.22	−.01	.15	–

DESM: Descriptive Experience Sampling Method; OCI-R: Obsessive Compulsive Inventory–Revised; AQ-10: Autism Quotient-10.

* *p* < .05. **p *<* .01. ***p *<* .001.

In the autism group, individuals who reported more positive thoughts during the DESM phase of the study scored lower on insistence on sameness *r* = −.42, *p* < .01, obsessive-compulsive features *r* = −.36, *p* < .05, obsessing *r* = −.32 *p* < .05 and the autism quotient *r* = −.35, *p* < .05, but these associations were not found in the control group. Autistic individuals who reported higher insistence on sameness reported significantly higher scores on the obsessive-compulsive inventory *r* = .67, *p* < .001, obsessing *r* = .46 *p* < .01 and the autism quotient *r* = .50, *p* < .001. Autistic participants who reported more obsessing had significantly fewer repeated thoughts *r* = −.31 *p* < .05, unlike the control group. Similarly to the control group, autistic participants reporting more obsessing had higher insistence on sameness and higher overall OCI-R scores *r* = .65 *p* < .001.

In the control group, individuals who reported more positive thoughts also reported significantly more visual thoughts *r* = .29, *p* < .05. Similarly to the autistic participants, in control participants higher insistence on sameness were associated with significantly higher obsessive-compulsive scores *r* = .67, *p* < .001, and autism quotient scores, *r* = .34, *p* < .01. Furthermore, control participants who reported more obsessing had higher insistence on sameness *r* = .32, *p* < .01, and higher overall OCI-R scores *r* = .75, *p* < .001. Unlike autistic participants, control participants who reported higher obsessive-compulsive scores had significantly higher scores on the autism quotient, *r* = .41, *p* < .01.

## Discussion

We aimed to understand repetitive and visual thinking in autism. We found tentative support for our hypothesis that autistic people would report experiencing repeated thoughts more frequently than non-autistic people. Autistic participants reported a similar number of thought categories or types of thoughts to non-autistic participants. We did not find support for our hypotheses that thinking would be more negative and more visual in autistic participants compared with controls. Finally, our hypothesis that repetitive thinking would predict repetitive behaviour was not supported.

Our finding that autistic people reported more repeated thoughts than controls during the DESM part of this study fits with the assumption that the repetitive behavioural profile found in autism extends to cognition. It is consistent with findings of higher levels of rumination, that is, repetitive thinking about distress, in autistic people compared with controls in other studies (e.g. Crane et al., 2013; Gotham et al., 2014). However, we did not find evidence that self-ratings of thoughts were more negative in the autism group. It may be that thinking is not more negative in autistic individuals, or that our measure of thoughts being ‘positive’ or ‘negative’ was too blunt to pick up the range of emotional experiences in participants. This finding could also be linked to alexithymia, which has been reported in autism and is characterised by difficulties in accessing and reporting one’s emotional experience (Kinnaird et al., 2019). We did not find evidence for restricted thinking style in our sample, and perhaps this is because autistic people do not have internal, cognitive restriction alongside restricted behaviours. It is also possible that this was due to inaccurate measurement as the average number of categories used in both groups was just under 5, the maximum number of categories used. Moreover, we defined restricted thinking using thought categories which were coded by non-autistic researchers, which may have reduced the relevance of our codebook. In autism, more idiosyncratic categories might be more relevant when coding restricted thinking.

Autistic participants in this study did not report experiencing a higher proportion of visual thoughts than control participants. This is in contrast to the findings of Hurlburt et al. (1994). This does not necessarily mean that visual aides are not still useful for autistic individuals; indeed there is evidence to support the efficacy of a wide range of interventions which use visual information for autistic people, particularly in children and young people. For example, social stories, which use words and pictures to teach autistic children how to navigate new situations, have been found to be effective (Karkhaneh et al., 2010). In the present study, it was not feasible to both recruit our target sample size and employ mixed methods. Future studies are needed with larger sample sizes and that use mixed methods as in Hurlburt’s original study to further investigate the autistic experience of cognition.

Autistic participants who reported more obsessional thoughts also reported significantly higher levels of insistence on sameness, but we did not find a relationship between our DESM measure of repetitive thinking and insistence on sameness. The OCI-R obsessive thinking subscale focuses on ego-dystonic negative thoughts that cannot be controlled, suggesting that these may be related to some insistence on sameness behaviours. It is also possible that there is some overlap at an item-level between the measures. We also found an unexpected negative association between obsessive thinking and repetitive thinking in autistic participants. It is possible that participants were only reporting thoughts that they experienced as positive, and not reporting obsessional thoughts in the DESM, leading to a negative correlation between these two measures. These findings suggest that anxiety, specifically obsessive thoughts, may be driving insistence on sameness more than generic repetitive thinking. In autistic children, insistence on sameness has been found to be associated with anxiety (Russell et al., 2019; Wigham et al., 2015). Other research has demonstrated that children with higher insistence on sameness had lower inhibitory control (Bos et al., 2019) and associations between executive function, insistence on sameness and anxiety (Uljarević et al., 2017). Future research to unravel the associations between inhibitory control and other executive functions, alongside insistence on sameness and anxiety in autistic adults, is warranted.

A strength of our study was the use of an adapted DES to support autistic adults to report their cognitions. The method was feasible, and participants were able to use the alert system to notice and report thought form and content in the moment, allowing us to gain insights into the internal experience of autistic people, as in previous studies (Chen et al., 2016; Hare et al., 2016; Hintzen et al., 2010; Kovac et al., 2016). Using our coding system, there were very few differences in the thoughts reported by autistic and non-autistic people. This extended to thoughts that were categorised as metacognitive, that is, thoughts which represented reflective and introspective cognitions.

A potential limitation of this study is that we sorted groups based on having a validated diagnosis of autism only. The AQ-10, while helpful as a screening tool and a means of characterising participants, does not have diagnostic validity (Ashwood et al., 2016) and studies have raised questions about the reliability of this measure in capturing characteristics of autism that do not reach clinical significance (Bertrams & Shah, 2021). To exclude or include participants on the basis of AQ-10 scores would have significant limitations as a method. Another limitation is the relatively small number of DESM samples collected, as well as imbalanced groups with both having a high number of females. In this study, we aimed for a breadth over depth, with a larger number of participants completing a smaller number of DESM samples. Furthermore, due to funding restraints, we used paper booklets along with a random alarm, rather than using an app or personal digital assistant, whereby it would have been possible to guarantee that records were completed contemporaneously. While the DESM method was feasible and cost-effective, we cannot be confident for either group that thoughts were reported as they occurred ‘in the moment’. However, we did maintain contact with participants during the 5-day period, and participants appeared engaged and motivated to report thoughts as directed in the comprehensive training session. Furthermore, participants might have filtered or censored their reporting according to social desirability, a need to preserve privacy or internal distress. This could account for the unexpected negative correlation between obsessional thoughts and repetitive thoughts, for example, unwanted, intrusive obsessional thoughts were more likely be suppressed and not reported. The lack of difference in visual thoughts reported might be accounted for by our method of collecting this data, people might have been more reticent or less confident or able to draw in the DESM booklets, preferring to ‘translate’ the images or pictures into words. They might have taken the visual image question literally, so even if the thought was experienced as an image, perhaps because they wrote it in words, it was reported as a verbal thought. Finally, researchers coding the booklets and making contact with participants for the duration of the study were aware of the hypotheses and may have exerted influence on the findings; this was mitigated by double-rating some of the DES records and by having multiple rounds of recruitment with different researchers.

This study investigated the cognitive experience in autism, and autistic participants reported higher rates of repetitive thinking compared with controls when measured using DES. Contrary to our hypothesis, we did not find evidence for more restricted, more negative, and more visual thinking in autistic participants. Obsessive thinking was associated with behavioural repetition, while repetitive thinking was not. The phenomenology of anxiety in autism and its relationship to behavioural repetition merits further exploration.
